# Hypothesizing the Biotherapeutic Potential of *Nitrosotalea devanaterra*: Targeting Ammonia Dependency to Disrupt *Helicobacter pylori* Survival Strategies in Gastritis

**DOI:** 10.5152/eurasianjmed.2026.251203

**Published:** 2026-01-16

**Authors:** Mohamad Warda, Mehmet Cemal Adıgüzel, Samet Tekin, Fikret Çelebi, A. M. Abd El-Aty

**Affiliations:** 1 Department of Physiology, Atatürk University Faculty of Veterinary Medicine, Erzurum, Türkiye; 2 Department of Biochemistry and Molecular Biology, Cairo University Faculty of Veterinary Medicine, Giza, Egypt; 3 Department of Microbiology, Atatürk University Faculty of Veterinary Medicine, Erzurum, Türkiye; 4 Department of Pharmacology, Cairo University Faculty of Veterinary Medicine, Giza, Egypt; 5 Department of Medical Pharmacology, Atatürk University Faculty of Medicine, Erzurum, Türkiye

**Keywords:** Ammonia competition, biotherapy, gastric microbiome, *Helicobacter pylori*, Mongolian gerbil model, *Nitrosotalea devanaterra*

## Abstract

The increasing antibiotic resistance of *Helicobacter pylori* (*H. pylori*) underscores the urgent need for alternative, nonantibiotic therapeutic strategies. This conceptual framework hypothesizes that *Nitrosotalea devanaterra* (*N. devanaterra*), an ammonia-oxidizing acidophile, could function as a biological competitor to *H. pylori* by reducing local ammonia availability, a critical factor for its survival and colonization in the gastric environment. To explore this hypothesis, a stepwise experimental framework is proposed. Initially, in vitro coculture models using gastric epithelial cells under microaerophilic conditions were employed to investigate potential interactions, metabolic competition, and impacts on *H. pylori* viability. Prospective in vivo validation could subsequently be performed using Mongolian gerbils, a model that closely mimics human gastric physiology, to assess the microbial load, histopathological changes, and host immune responses under controlled conditions. While direct empirical evidence for *N*. *devanaterra *survival and activity in the highly acidic gastric milieu is currently lacking, preliminary theoretical analysis suggests that ammonia competition could influence *H. pylori* persistence and pathogenesis. This hypothesis-driven approach emphasizes a microbiome-inspired strategy that does not rely on antibiotics, potentially reducing selective pressure for resistance. By introducing the concept of targeted nutrient competition as a therapeutic modality, this framework aims to stimulate further research into the feasibility of employing environmental acidophiles as modulators of pathogenic bacteria in the stomach. The proposed strategy provides a foundation for future studies evaluating *N. devanaterra* or related ammonia-oxidizing microorganisms as innovative, nonantibiotic interventions against *H. pylori* infection.

**Main Points**

- Concept: This study proposes the use of *Nitrosotalea devanaterra*(* N. devanaterra *), an ammonia-oxidizing acidophile, as a biotherapeutic agent to combat *Helicobacter pylori* (*H. pylori*)by depleting ammonia, which *H. pylori* needs to survive stomach acidity.
- Method: A multiphase approach is proposed—starting with in vitro coculture of gastric cell lines, followed by in vivo testing in Mongolian gerbils—to assess microbial competition, ammonia levels, and host immune responses.
- Goal & Challenges: If effective, this could offer nonantibiotic treatment for gastritis and antibiotic-resistant *H. pylori*, although the survival of *N. devanaterra* in gastric acid and safety concerns require further study.

## Introduction

*Helicobacter pylori* (*H. pylori*) is a gram-negative bacterium that colonizes the gastric mucosa and contributes to gastritis, peptic ulcers, and gastric carcinoma.^1^ Its ability to withstand extreme gastric acidity is supported by urease-mediated neutralization and adherence to the gastric epithelium.^2^^,^^3^ The increasing antibiotic resistance rates worldwide^4^ highlight the urgent need for alternative therapeutic strategies, including microbial competition and microbiome-based interventions.^5^

In recent years, studies have indicated that probiotics can compete with the growth of *H. pylori*.^6^ *Nitrosotalea devanaterra* (*N. devanaterra*) may inhibit *H. pylori* growth by competing for ammonia, a key nutrient in gastric colonization. However, its mechanisms of action and interactions with *H. pylori* in the gastric environment remain unclear. Clarifying these dynamics, particularly in relation to gastric epithelial cells, could guide the development of novel microbiome-based strategies for *H. pylori* management.

In this context, several probiotic and microbiome-based strategies—most notably Lactobacillus and Bifidobacterium species—have been explored for *H. pylori* management through mechanisms, such as competitive adhesion, microenvironment acidification, or the production of antimicrobial metabolites. These approaches differ fundamentally from the ammonia-depletion mechanism proposed here for *N. devanaterra*. To situate this hypothesis within existing work, Table 1 provides a concise comparison of representative microbiome-based strategies and their modes of action.

The gastric milieu poses formidable obstacles to bacterial viability, exacerbated by conditions of intense acidity and enzymatic activity.^3^ Adaptation to such hostile environments, as exemplified by *N. devanaterra*, demands profound acclimatization strategies. Should these adaptive measures prove inadequate, researchers may explore strategies such as increasing bacterial loads or optimizing growth conditions to increase survival rates during experimental investigations. These efforts are crucial for advancing the understanding of bacterial behavior in the gastrointestinal tract and improving therapeutic interventions against microbial infections.

Furthermore, the concept of cocultivating *N. devanaterra* with *H. pylori* in simulated gastric media could provide critical information on how these 2 bacteria interact and potentially inhibit *H. pylori* growth. This competition could lead to a deeper understanding of the microbial dynamics within the stomach and of how nonpathogenic bacteria can be leveraged to control or even prevent pathogenic colonization.

The complex relationship between gastric bacteria and the host environment also extends beyond the microbial community to involve host immune responses.^7^ Coculturing *N. devanaterra* with *H. pylori* on gastric cell lines (AGS, GES-1, and RGM-1) may elucidate bacterial interactions and host responses. Moreover, the Mongolian gerbil (*Meriones unguiculatus*), recognized as the most reliable in vivo model for *H. pylori* research,^8^ provides an optimal system for evaluating the therapeutic potential of *N. devanaterra* in mitigating *H. pylori*-induced gastric inflammation and pathology.

This study aims to evaluate the potential of *N. devanaterra* to inhibit *H. pylori* growth through ammonia competition and assess its viability as a microbiome-based therapeutic strategy. These findings are expected to provide foundational evidence for the development of innovative, nonantibiotic interventions against *H. pylori* infection amid the escalation of antibiotic resistance.

Although *N. devanaterra* has not been evaluated in gastric environments, several biochemical traits support its theoretical relevance to an ammonia-competition strategy against *H. pylori*. As an obligate ammonia-oxidizing acidophile, *N. devanaterra* exhibits exceptionally high-affinity ammonia uptake, robust ammonia monooxygenase activity, and physiological adaptations to low-pH habitats—including proton-resistant membrane lipids, acid-stable enzymes, and pH-homeostasis mechanisms. These characteristics suggest that, at least conceptually, *N. devanaterra* could transiently function as an ammonia-depleting competitor and thereby influence *H. pylori* survival. While empirical validation is still needed, outlining these biochemical capacities clarifies the mechanistic rationale underlying this hypothesis.

As the viability of *N. devanaterra* under gastric-like acidity remains untested, its key physiological and biochemical traits relevant to ammonia oxidation in acidic microenvironments were outlined. These traits provide a mechanistic context for the proposed ammonia- competition with *H. pylori* while underscoring the absence of empirical data specific to the gastric niche.

The biochemical traits summarized in Table 2 provide the mechanistic basis for considering *N. devanaterra* as a potential ammonia competitor, as they collectively indicate efficient low-pH ammonia oxidation, an acid-tolerant membrane and enzymatic adaptations, and a strict reliance on ammonia as an electron donor. However, none of these properties have been evaluated under gastric conditions, and there is currently no evidence that *N. devanaterra* can survive or remain metabolically active at gastric pH levels. Accordingly, its role in the stomach should be considered a conceptual possibility that requires direct experimental verification. Although the present study does not include experimental analyses under gastric-like conditions, it is theoretically plausible that *N. devanaterra* could be progressively adapted to tolerate lower pH values and select components of gastric fluid through controlled acclimation strategies. Such adaptive approaches—if successful—might help the organism withstand not only increased acidity but also limited exposure to gastric enzymes such as pepsin, which together define the biochemical constraints of the gastric niche. This possibility remains strictly hypothetical and requires systematic, stepwise evaluation; however, the conceptual feasibility of physiologically guided adaptation provides a rational foundation for future studies aimed at determining whether *N. devanaterra* can achieve short-term viability or retain ammonia-oxidizing activity under gastric-relevant chemical conditions.

## Material and Methods 

### Study Design

*Helicobacter pylori* infection is a major etiological factor in peptic ulcers and gastric cancer, with treatment challenges exacerbated by increasing antibiotic resistance.^16^^,^^17^ Microbial competition is increasingly recognized as influencing *H. pylori* colonization dynamics.^18^^,^^19^ It was proposed that *N. devanaterra*, an acidophilic ammonia oxidizer, may act as a biological competitor by reducing ammonia availability, thereby inhibiting *H. pylori* growth and survival.^5^ Since the work is hypothetical, ethical approval for the present work is not applicable.

### Ethical Statement

Since this work presents a conceptual and hypothesis-based framework without the use of human participants or animal experimentation, ethical approval is not applicable to the present study.

### I*n Vitro* Coculture Experiments

To test this hypothesis, *H. pylori* can be cocultured with *N. devanaterra* in gastric epithelial cell lines, including AGS, GES-1, and RGM-1, under microaerophilic conditions simulating gastric acidity.^18^ Bacterial growth was assessed via optical density and colony-forming unit (CFU) counts.^19^

Ammonia concentrations in the culture medium were measured to determine whether *N. devanaterra* depletes this nutrient, which is essential for *H. pylori* metabolism. Host responses, including cytotoxicity, confluence, and cytokine production, can be evaluated to assess the impact of microbial competition on epithelial health.

### Molecular and Metabolic Analyses

Gene expression profiling is proposed to investigate how *N. devanaterra* may affect *H. pylori* ammonia metabolism, virulence factor expression, and stress responses via RNA-Sequence and quantitative polymerase chain reaction validation.^20^^,^^21^ Metabolomic analyses using nuclear magnetic resonance spectroscopy are suggested to compare coculture versus monoculture metabolic profiles.^22^^,^^23^ Fluorescence in situ hybridization (FISH) and confocal microscopy can be used to visualize spatial distribution, biofilm architecture, microbial interactions,^24^ and direct antagonistic interactions. To investigate these processes, FISH will be used to visualize the spatial distribution and competition in cocultures.^25^ Next-generation sequencing can be used to assess interspecies interactions and relative abundance shifts.^26^

### Growth Inhibition and Biofilm Disruption Assays

It is hypothesized that *N. devanaterra* may inhibit *H. pylori* growth and biofilm formation.^27^ The proposed assays include optical density and CFU counts for growth inhibition and crystal violet staining combined with microscopy to assess biofilm disruption.^28^ Additionally, *N. devanaterra* may interfere with *H. pylori* biofilm architecture and virulence, limiting its pathogenic potential.^24^ Significant impairment of growth, biofilm stability, or virulence would provide functional evidence supporting *N. devanaterra* as a potential biotherapeutic agent.

### *In Vivo* Validation Using Mongolian Gerbils

Male Mongolian gerbils (*Meriones unguiculatus*), considered the most reliable animal model for *H. pylori* research, are proposed for in vivo validation. Gerbils were inoculated with *H. pylori*, *N. devanaterra*, or both. Gastric colonization was quantified in tissue and fecal samples. Histopathological evaluation can be used to assess inflammation and epithelial integrity, whereas cytokine profiling (e.g., TNF-α and IL-6) and immune cell infiltration can be used to measure immune modulation.^8^ Clinical outcomes, including weight loss and gastric distress, were monitored. These experiments aimed to determine whether *N. devanaterra* can outcompete *H. pylori*, reduce pathology, and modulate host immune responses.

### Data Analysis

The data will be analyzed to compare bacterial loads, biofilm formation, ammonia concentrations, cytokine levels, and histopathology across the experimental groups. Evidence of reduced *H. pylori* growth or pathology in the presence of *N. devanaterra* would support the concept of competitive exclusion as a microbiome-inspired therapeutic strategy.

## Conclusion

*N. devanaterra*, an acidophilic bacterium capable of surviving in highly acidic environments, represents a promising biotherapeutic candidate against *H. pylori*. By competing for essential nutrients such as ammonia, *N. devanaterra* may reduce *H. pylori* colonization, mitigate gastritis, and support gastric mucosal integrity. If validated, this approach could offer a microbiome-based alternative to antibiotics, potentially limiting antimicrobial resistance. While preliminary studies in gastric cell lines and animal models are encouraging, further research is needed to clarify interactions with *H. pylori*, host immunity, and the gastric microbiome, as well as to assess translational feasibility in humans.

### Limitations, Safety Considerations, and Future Directions

While this study proposes *N. devanaterra* as a potential microbiome-based competitor of *H. pylori*, several limitations must be acknowledged. First, the survival and activity of *N. devanaterra* in gastric conditions below pH 4.0 remain unproven, and its efficacy in outcompeting *H. pylori* has not yet been empirically validated. Second, the introduction of a nonnative organism carries ecological and safety risks, including possible disruption of the gastric microbiota, unintended immune modulation, or unforeseen host–microbe interactions. Third, while the Mongolian gerbil is a reliable *in vivo* model for *H. pylori* research, findings may not fully extrapolate to human physiology, necessitating future studies using advanced models such as gastric organoids or primates. Despite these challenges, this exploratory framework establishes a foundation for further investigations, including rigorous in vitro and in vivo validation of ammonia competition, safety profiling, microbiome analysis, and eventual translational studies. Ultimately, these studies aimed to clarify the therapeutic potential and feasibility of *N. devanaterra* as a nonantibiotic intervention for *H. pylori*-related gastric diseases.

#### Declaration of Generative AI and AI-assisted Technologies in the Writing Process

During the preparation of this work, the authors used ChatGPT (GPT4; OpenAI, 2024) for language editing. After using this tool, the authors reviewed and edited the content as needed and take full responsibility for the content of the publication.

## Figures and Tables

**Table 1.** Probiotic Mechanisms and Outcomes Against *H. pylori*

Strategy/Organism	Proposed Mechanism (Corrected and Evidence Based)	Reported Outcome	Key References
*Lactobacillus spp.*	Competitive adhesion; lactic acid production lowering gastric pH; bacteriocin release; suppression of IL-8 and other pro-inflammatory cytokines; interference with *H. pylori* virulence factors	Reduced *H. pylori* load; improved gastritis symptoms; enhanced eradication when combined with therapy	^6^
*Bifidobacterium spp.*	Immunomodulatory cytokine modulation (↓ IL-8, ↓ TNF-α, ↑ IgA); antimicrobial effects reducing *H. pylori* colonization; competition for gastric/intestinal adhesion sites	Reduced *H. pylori* load; decreased gastric inflammation and mucosal damage	^7^
*Bacillus spp.* (e.g., *B. subtilis*)	Production of antimicrobial peptides; disruption of biofilms; immunomodulation (↑ IL-10, ↓ IL-8); high survivability in gastric conditions	Decreased colonization and virulence; reduced inflammation	^8^

**Table 2.** Biochemical and Physiological Traits of *N. devanaterra* Relevant to Its Proposed Role as an Ammonia Competitor to *H. pylori*. The Table Summarizes the Properties Reported From Environmental and Genomic Studies; None of These Traits Have Been Experimentally Evaluated Under Gastric Conditions

**Trait/Parameter**	**Reported Property**	**References**
Habitat/ecology	Acidophilic AOA isolated from low pH soils	^5,^^9,^^10^
pH tolerance	Activity documented at pH ~4.0-5.0; obligatory acidophilic	^5,^^9,^^10^
Ammonia uptake affinity	High-affinity ammonia oxidation; efficient at low substrate concentrations	^11^
Key enzymes	Ammonia monooxygenase (AMO) and downstream hydroxylamine oxidation pathways	^12,^^13^
Membrane/adaptive features	Archaeal ether lipids; traits associated with proton resistance and acid stability	^9-^^11,^^14,^^15^
Metabolic lifestyle	Obligate chemolithoautotroph; relies exclusively on ammonia as electron donor	^5,^^11^
Known ecological constraints	Slow growth rates; oxygen requirement; adaptation to soil niche	^5,^^11^
Relevance to gastric setting	Conceptual potential to deplete local ammonia if transiently active; empirical gastric viability not yet demonstrated	^5,^^11^

AOA, Ammonia oxidizing archaea.

## Data Availability

Not applicable since no data were collected.
